# A Crosstalk Between *Brachypodium* Root Exudates, Organic Acids, and *Bacillus velezensis* B26, a Growth Promoting Bacterium

**DOI:** 10.3389/fmicb.2020.575578

**Published:** 2020-10-06

**Authors:** Meha Sharma, Dina Saleh, Jean-Benoit Charron, Suha Jabaji

**Affiliations:** Department of Plant Science, Faculty of Agricultural and Environmental Sciences, McGill University, Montreal, QC, Canada

**Keywords:** TCA cycle, biofilm, root exudate component, *Brachypodium distachyon*, chemotaxis, GC-MS

## Abstract

Plant growth-promoting rhizobacteria (PGPR) are associated with plant roots and use organic compounds that are secreted from root exudates as food and energy source. Root exudates can chemoattract and help bacteria to colonize the surface of plant roots by inducing chemotactic responses of rhizospheric bacteria. In this study, we show that root colonization of *Brachypodium distachyon* by *Bacillus velezensis* strain B26 depends on several factors. These include root exudates, organic acids, and their biosynthetic genes, chemotaxis, biofilm formation and the induction of biofilm encoding genes. Analysis of root exudates by GC-MS identified five intermediates of the TCA cycle; malic, fumaric, citric, succinic, oxaloacetic acids, and were subsequently evaluated. The strongest chemotactic responses were induced by malic, succinic, citric, and fumaric acids. In comparison, the biofilm formation was induced by all organic acids with maximal induction by citric acid. Relative to the control, the individual organic acids, succinic and citric acids activated the *epsD* gene related to EPS biofilm, and also the genes encoding membrane protein (*yqXM*) and hydrophobin component (*bslA*) of the biofilm of strain B26. Whereas *epsA* and *epsB* genes were highly induced genes by succinic acid. Similarly, concentrated exudates released from inoculated roots after 48 h post-inoculation also induced all biofilm-associated genes. The addition of strain B26 to wild type and to *icdh* mutant line led to a slight induction but not biologically significant relative to their respective controls. Thus, B26 has no effect on the expression of the *ICDH* gene, both in the wild type and the mutant backgrounds. Our results indicate that root exudates and individual organic acids play an important role in selective recruitment and colonization of PGPR and inducing biofilm. The current study increases the understanding of molecular mechanisms behind biofilm induction by organic acids.

## Introduction

Plant-growth promoting rhizobacteria (PGPR) are soil and rhizosphere-derived inhabitants that colonize plant roots and positively influence plant growth and augment immunity (Benizri et al., 2001; Gaiero et al., 2013). It is apparent that effective colonization of the root system is essential for the exertion of beneficial effects of PGPR (Hardoim et al., 2008; Lareen et al., 2016). One of the plant’s determinants affecting microbial communities in the rhizosphere is root exudates (Badri and Vivanco, 2009). Abundant amounts of photosynthates of low and high molecular weight compounds are secreted as root exudates into the rhizosphere. Root exudation includes organic acids, enzymes, phenolics, sugar and carbohydrates (mucilage) and proteins (Hawes et al., 2000; Bais et al., 2004). In the rhizosphere, these complex organic compounds may serve as chemoattractants or chemorepellents for plant-beneficial microbes (Badri and Vivanco, 2009; Liu et al., 2017). Therefore, understanding the role of organic acids in root exudates in influencing the PGPR community structure and function is of paramount importance for plant development.

Organic acids are generated from intermediates of the tricarboxylic acid cycle (TCA) and are exuded into the rhizosphere (Ryan et al., 2001; Zhang and Fernie, 2018). These intermediates are synthesized by various enzymes viz; citrate from Citrate synthase (CS), fumarate from Succinate dehydrogenase (SDH), malate from Malate dehydrogenase (MDH), Fumarate hydratase (FH), succinate from Succinyl-CoA synthetase, and 2-Oxoglutarate from NADP-dependent Isocitrate dehydrogenase (ICDH) (Araujo et al., 2012). Organic acids are an essential driver of bacterial activity in the rhizosphere (Eilers et al., 2010). It is believed that root exudates take on a dialogue role between plants and rhizospheric microbes in the efficient recruitment of rhizospheric microbes (Bais et al., 2004; Sun et al., 2012). Several reports have documented the regulatory role of organic acids in plant-microbe interactions (Kamilova et al., 2006; Adeleke et al., 2017). Others demonstrated that specific organic acids released from plant roots can attract and recruit specifically single species of bacteria in the rhizosphere. For example, motility and chemotactic response of *Pseudomonas fluorescens* WCS365 toward tomato (*Solanum lycoperscum* L.) roots were induced by malic and citric acids (De Weert et al., 2002). Citric and malic acids secreted from *Arabidopsis thaliana* roots and watermelon (*Citrullus vulgaris* L.) roots attracted and enhanced root binding of *Bacillus subtilis* FB17 biofilm (Rudrappa et al., 2008), and also recruited *Paenibacillus* SQR-21 in the rhizosphere (Ling et al., 2011). However, the effect of PGPR on the expression of TCA cycle genes is still unknown. The first two elements of bacterial interaction with plant roots are the attraction of bacteria toward plant roots through chemotaxis (Gaworzewska and Carlile, 1982), leading to colonization and biofilm formation. Biofilm formation on the roots is indicative of successful plant-PGPR colonization. The biofilm matrix of endospore-forming *Bacillus* species is composed of exopolysaccharides (EPS), amyloid-like fibers, and the coat protein, biofilm-surface layer protein (BslA) which is composed of hydrophobin component (Branda et al., 2004, 2006; Cairns et al., 2014). EPS formation is controlled by 15-gene *epsA-O* operon (Branda et al., 2004). Among the eps operons, gene *epsA* and *epsB* are the membrane component of tyrosine kinase, which forms EPS (Elsholz et al., 2014). At the same time synthesis of fibers is controlled by three gene *tapA-sipW-tasA* operon. However, for the delivery of the biofilm matrix component protein (TasA), another gene *yqxM*, is required (Lemon et al., 2008). Although several studies have explored plant-microbe interactions, few explored how root exudates regulate biofilm-associated genes.

It is well documented that *Bacillus* spp. stimulate plant growth by increasing nutrient availability through the synthesis of phytohormones, or suppressing plant diseases (Chauhan et al., 2019). We demonstrated that *B. velezensis* B26, previously known as *B. methylotrophicus* strain B26, internally colonized the model plant *Brachypodium distachyon* and accelerated its growth by the production of phytohormones, volatiles and various antimicrobial compounds (Gagné-Bourgue et al., 2013; Gagné-Bourque et al., 2015). Furthermore, exposure of inoculated *Brachypodium* and timothy grass to extended drought conditions improved their tolerance to drought stress by increasing the accumulation of either acquired or inducible osmolytes associated with drought protection compared to non-inoculated plants (Gagné-Bourque et al., 2015, 2016).

However, the role of *Brachypodium* root exudates and the interaction of root colonization with strain B26 are yet to be established. In the present study, the objectives were to determine whether exogenously-added organic acids and GC-MS identified organic acids released from roots of B26-colonized *Brachypodium* could (i) promote the expression of *Brachypodium* genes encoding the respective organic acids in the TCA cycle; (ii) induce chemotactic responses of strain B26; (iii) promote the biofilm formation of B26 by activating the expression of biofilm-associated genes, and that (iv) strain B26 could alter the expression of *Brachypodium* mutant lines overexpressing organic acid genes relative to the colonized wild type. This study is useful to understand the role of root exudates in plant-PGPR interactions.

## Materials and Methods

### Bacterial Strain and Culture Conditions

*Bacillus velezensis* strain B26 (GenBank Accession number LGAT00000000) formally known as *B. methylotrophicus*strain B26 (Gagné-Bourgue et al., 2013; Jeukens et al., 2015), was originally isolated from leaf blades and seeds of the bioenergy crop switchgrass (*Panicum virgatum* L.). The strain B26 was stored in Lysogeny Broth (LB) (BDH chemical Ltd., Mississauga, ON, Canada) supplemented with 20% glycerol at −80°C and recovered on LB at 28 ± 1.0°C on a rotatory shaker at 120 rpm overnight. Following appropriate dilution in LB broth, 10^8^ CFU.mL^–1^ (OD_600_ of 1.0) were used in all experiments unless otherwise stated.

### Plant Material and Growth Conditions

Seeds of *Brachypodium* wild type Bd21-3 and T-DNA mutant lines (Table 1) with overexpression or loss of function were sourced from the DOE Joint Genome Institute, CA^1^. Seeds of *B. distachyon* wild type accession Bd21-3 were soaked overnight in sterile distilled water at room temperature, after which the lemma was removed. The seeds were sterilized in 70% ethanol for 30 s, 1.3% sodium hypochlorite for 4 min, and then rinsed three times with sterile distilled water (Vain et al., 2008). For stratification and vernalization, sterile seeds were placed between two sterile filter papers moistened with sterile distilled water in a Petri dish incubated at 4°C in the dark for 15 days.

Multiplication of *Brachypodium* mutant lines was performed following the protocol of DOE Joint Genome Institute, CA^2^. Briefly mutant seeds were sown in pots (6.35 × 6.35 × 7.62 cm) containing G2 Agro Mix^®^ (Plant Products Co., Ltd.) and were watered to field capacity. Pots were wrapped with cling film and aluminum foil to preserve the moisture and block any source of light, and were left undisturbed to allow vernalization at 4°C in the dark. A week later, pots were placed in a growth chamber set under the following conditions: 16 h photoperiod, 150 μmoles.m^2^. s^–1^ of light intensity, and day/night temperature of 25°C/23°C.

### Genotyping of T-DNA Mutants

Multiplex PCR was performed using Gene Specific Primers (GSP) and T3 T-DNA left border primer. PCR-based genotyping was carried out on T-DNA mutant lines (Table 2) to screen for homozygous plants. Primer sets were designed based on gene sequences retrieved from Phytozome Bd21-3 v1.1 genome (Phytozome v12.1^3^). Primer 3 web tool (version 4.0.0) was used to design gene specific primers (GSP) from at least 500 bases on either side of the putative insertion site in the gene (Table 2). Genotyping of all mutant lines was performed following the protocol of DOE Joint Genome Institute, CA^4^. Seeds of homozygous mutant lines JJ19999 overexpressing *Isocitrate dehydrogenase* (*ICDH*) were retained for downstream applications. Genotyping of the remaining T-DNA-mutant lines did not yield homozygous lines even after two generations.

### Root Exudate Collection

#### Experiment 1

Semi-*hydroponic*s system (Supplementary Figure S1) was developed for the collection of root exudates using Magenta GA-7 tissue culture boxes (7.62 × 7.62 × 10.2 cm) (Sigma-Aldrich, United States). Pre-germinated seeds of wild type Bd21-3 (5 seeds/box) were transferred to Magenta boxes filled with an inert substrate consisting of a mixture of 1.7–2.5 mm sterile glass beads of low alkali (Ceroglass, United States) up to 2 cm in height. Beads were saturated with 1/4 strength Hoagland’s solution (pH 6.0, buffered with 2 mM MES (2-[N-morpholino] ethanesulfonic acid). Glass beads supported good root mass (Supplementary Figure S1). A total of 12 magenta boxes were used in this experiment, and each box represents an experimental unit. Boxes were transferred to a growth cabinet (Conviron, Canada) with light intensity of 150 μmoles. m^2^.s^–1^ 16 h light and 8 h dark at day/night temperatures of 25°C/23°C. After 40 days of growth, six boxes were inoculated with 500 μl of B26 (OD_600_ of 1) inoculum suspended in phosphate buffer (1 M, pH 7) and six control boxes received 500 μL of phosphate buffer alone. All boxes were incubated in a controlled growth cabinet under the previously described conditions. After 48 h, inoculated and control *Brachypodium* seedlings from every two experimental units were pooled (total of 10 seedlings) to make 3 biological replicates per treatment. Prior to exudate collection, roots of intact plants from each replicate were rinsed off once in 20 mL of ultra-pure water for 2 h to remove cell debris and nutrient solution. The root system was placed in a 150 mL glass beaker so that the roots were fully immersed in 20 mL of ultra-pure water with gentle agitation for 24 h under the same growth chamber conditions (Supplementary Figure S1)The solution (20 mL) was filter-sterilized using 0.22 μm filter, freeze-dried, concentrated at 50x in ultra-pure sterile water and stored at −20°C for downstream applications. Portions of the inoculated and control roots were processed prior and after root exudate collection for the visualization of B26 biofilm using scanning electron microscopy. The presence of B26 on and inside roots prior and after root exudate collection was confirmed by PCR. The remaining of the roots of each treatment were immersed in liquid nitrogen and stored at −80°C for mass spectrophotometric analysis and gene transcription of organic acids.

#### Experiment 2

To explore the possible role of plant-derived organic acid genes in chemotactic response and colonization of *Brachypodium* by strain B26, comparative transcript expression was measured between roots of wild type Bd21-3 and homozygous *Isocitrate dehydrogenase (icdh)* T-DNA mutant line (Table 1)^5^. Pregerminated seeds of wild type Bd21-3 and *icdh* mutant were grown in semi-hydroponic Magenta boxes. Each Magenta box had 5 seeds of wild type or *icdh* mutant, and were grown under controlled conditions as described above. Three Magenta boxes represented biological replicates for wild type and mutant lines. Following 40 days of growth, both wild type and mutant lines were inoculated with strain B26. Roots were harvested 48 h post-inoculation, immersed in liquid nitrogen for downstream gene expression studies.

**TABLE 1:** Organic acid T-DNA mutant lines of *Brachypodium.*

Gene	T-DNA line	Gene tagged	Construct	Insert class	No. of homozygous mutants identified	Type of mutation	References
*Malate dehydrogenase*	JJ27103	Bradi3g12460	pJJ2LBA	Exon	NA	Overexpression due to activation tagging	Hsia et al., 2017
*Succinate dehydrogenase*	JJ11635, JJ11665, JJ11645,JJ11605, JJ11687, JJ11574, JJ11675, JJ11621	Bradi3g13980	pJJ2LBA	5′UTR	NA	Overexpression due to activation tagging	Hsia et al., 2017
*Citrate synthase*	JJ2510	Bradi3g08910	pJJ2LB	Exon	NA	Loss of function due to Insertional mutation	Bragg et al., 2012
*Isocitrate dehydrogenase*	JJ19999	Bradi2g45420	pJJ2LBA	Near*	4	Overexpression due to activation tagging^$^	Hsia et al., 2017

* Near means within 1,000 bp of the 3′ or 5′ end of the gene. ^$^Activation tagging construct is designed to increase the transcription of nearby genes.

**TABLE 2:** List of primers used in this study.

Gene of interest	Primer name	Sequence (5*′*→3*′*)	Annealing temperature (°C)	Product size (bp)	Source
**Primers for insertion mutation detection**					
*Citrate synthase*	CS-IN-F	CTGAGGCATTACACCCCTGT	56	427	BdiBd21-3.3G0119500.1
	CS-IN-R	TTCAGCAGTGAGAAGCCAGA			
*Malate dehydrogenase*	MD-IN-F	AAAAATGGGGCAGATCATCA	56	443	BdiBd21-3.3G0165100.1
	MD-IN-R	CATTGCAGGGTCGGTTACTT			
*Succinate dehydrogenase*	SD-IN-F	TGTCTTTCATGCGATTCAGC	56	480	BdiBd21-3.3G0184500.1
	SD-IN-R	CACCTGGAAGGAGGAATGAA			
*Isocitrate dehydrogenase*	ID-IN-F	ACTAATGGCGGATCTGA	56	496	BdiBd21-3.2G0578900.1
	ID-IN-R	GGTTCCCGGTGTTTGATTTA			
Hygromycin	Hyg-F	ATGAAAAAGCCTGAACTCACCGCGAC	58	950	Vogel Lab
	Hyg-R	CTATTTCTTTGCCCTCGGACGAGTGC			
*T-DNA left border primer*	T3 TDNA LB	AGCTGTTTCCTGTGTGAAATTG	56	120	Vogel lab
	R9 TDNA LB	GATAAGCTGTCAAACATGAGAATTCAG	56	120	Vogel lab
**qRT-PCR primers for organic acid genes**					
*Citrate synthase*	CS-Q-F	CTCCCGTCCTTCCTTCAAATAA	55	226	BdiBd21-3.3G0119500.1
	CS-Q-R	GATATCTAGAACCCGAGCAAGTC			
*Malate dehydrogenase*	RT-MD-F	TGCCAAGTGCTGTCCTAATG	55	171	BdiBd21-3.3G0165100.1
	RT-MD-R	AGCACTTCAGCCACAAAGGT			
*Succinate dehydrogenase*	SD-Q-F	CACGTCTTAGAAACCGCTGTA	60	112	BdiBd21-3.3G0184500.1
	SD-Q-R	CCCATGACTTCGCCCTTATT			
*Isocitrate dehydrogenase*	ID-Q-F	TACCCGTCATTTCCGTGTTC	60	92	BdiBd21-3.2G0578900.1
	ID-Q-R	TGTGTGCAAGTCCTCTTGTC			
*Fumarase hydratase*	FH-Q-F	GGTGAACTTAGCCTACCAGAAA	60	108	BdiBd21-3.1G0851300.1
	FH-Q-R	ACCCATAACCTGAGCACAAA			
*Succinyl coA synthetase alpha subunit*	LSC1-F	GGATCCTCAGACAGAAGGTATTG	60	131	BdiBd21-3.1G0308500.1
	LSC1-R	GTGCAGTAAGTCCAGCTATGAA			
*Succinyl coA synthetase beta subunit*	LSC2-F	GGAGGAACCAGCATTGAAGA	60	119	BdiBd21-3.3G0651500.1
	LSC2-R	GCCAGACCATCAACAACTTTAC			
*Ubiquitin*	UBC18-F	GGAGGCACCTCAGGTCATTT	60	193	Hong et al., 2008
	UBC18-R	ATAGCGGTCATTGTCTTGCG			
*Actin*	BdACTIN2-F	GTCGTTGCTCCTCCTGAAAG	55	188	Derbyshire and Byrne, 2013
	BdACTIN2-R	ATCCACATCTGCTGGAAGGT			
**Primers for biofilm associated genes**					
*epsA*	epsA-F	GCTGCGAAATATGGTCATGG	60	141	B26 Genome
	epsA-R	AGCGTCTGCTTCACTTTCTC			
*epsB*	epsB-F	CGCTCTATTCTCGTCACTTCTT	60	140	B26 Genome
	epsB-R	GTCTCGTGTATCGTCGGTTT			
*epsD*	epsD-F	GACAACGGCTACGACATGAT	60	102	B26 Genome
	epsD-R	GTACAGCACCTTTGTCCCTT			
*yqxM*	yqxM-F	ATTTTTACGGCTTTCGTTCATT	60	269	Xu et al., 2013
	yqxM-R	GTCCGCTCTTTTCCCTTATTCT			
*bslA*	bslA-F	CTGTCATGGCAAGTTTATTCGG	60	140	B26 Genome
	bslA-R	CTGGCTGGCACCTGTATATT			
*RecA*	recA-F	AAAAAACAAAGTCGCTCCTCCG	60	109	Xu et al., 2013
	recA-R	CGATATCCAGTTCAGTTCCAAG			

### Organic Acid Analysis Using GC-MS

#### Sample Preparation

All chemicals were of analytical grade and were purchased from Sigma-Aldrich (Oakville, ON, Canada). Freeze-dried root samples (30 mg) were pulverized into powder and lyophilized. Root exudates (116 μL) were transferred to 2 mL Eppendorf microtubes containing 200 μL of 80% methanol. There were three replicates for each treatment. For GC-MS analyses, the samples were sent to Rosalind and Morris Goodman Cancer Research Centre, McGill University, Quebec, Canada. Ceramic beads (32.8 mm) were added to the samples and processed in a homogenizer (Analytikjena SpeedMill Plus) for three times, 45 s each, followed by centrifugation at 4°C for 10 min at 1500 rpm. The supernatants were transferred to 1.5 mL Eppendorf tubes containing 1 μL of 800 ng.μL^–1^ of Myristic-d27 in pyridine and placed in a CentriVap vacuum centrifuge at 4°C for overnight drying. Myristic-d27 is an internal standard used for retention time-locking.

#### Sample Derivatization

Samples were derivatized by adding 30 μL of MOX (10 mg Methoxyamine: HCl per 1 ml anhydrous pyridine) to each sample. This methoximation converts unstable keto groups to stable methoxyamines. Samples were later derivatized with MTBSTFA(N-tert-butyldimethylsilyl-N-methyltrifluoroacetamide) to form the more volatile TBDMS (tert-butyldimethylsilyl) derivatives.

#### GC-MS Data Acquisition

GC-MS analyses were performed using Agilent 5975C mass selective detector coupled to a 7890A gas chromatograph (Agilent Technologies, Santa Clara, CA, United States) with 7693 autosampler and a DB-5MS DG capillary column (30 m plus 10 m Duraguard^®^) with a diameter of 0.25 mm, film thickness of 0.25 μm (Agilent J &W, Santa Clara, CA, United States) as described by Mamer et al. (2013). The GC-MS was run in electron ionization mode (70 eV) and Selected Ion Monitoring (SIM) mode. Data acquisition was done in Scan and SIM modes using MassHunter (Agilent) software. The spectra obtained were compared against the NIST (National Institute of Standards) database. The root samples had large amounts of malic and citric acids, and were diluted 1:40 before being run again in Scan mode. While root exudates did not require dilution. Data were represented as normalized area which is the area of peak divided by amount of sample in mg (roots) or μL (root exudates).

### Chemotaxis Assays

Organic acids including malic, citric, fumaric, and succinic produced by plants act as chemotactic agents to recruit beneficial bacteria to the rhizosphere (Tan et al., 2013), and could provide nutrients for microbial community in the rhizosphere, and act as chemo-attractants representing the initial step for microbial recruitment and colonization process (Sasse et al., 2018). The chemotactic response of strain B26 to organic acids was established using one quantitative method and two qualitative methods.

#### Method 1- Quantitative Chemotaxis Assay

A modified capillary chemotaxis assay was used to quantify the chemotaxis of B26 in response to different organic acids (Mazumder et al., 1999). The chemotactic system consisted of three components; a disposable 200 μL pipette tip as the chamber, a 251/8-gauge needle and 1 mL syringe as chemotaxis capillary. The syringe was filled with 500 μL of one of the following filter sterilized organic acids: malic, citric, fumaric, succinic and oxalic acids prepared at concentrations of 10, 25, and 50 μmol.L^–1^. Syringes with 500 μL sterile distilled water served as control for each of the chemoattractant. The needle–syringe capillary system was tightly inserted into the pipette tip containing 150 μL of bacterial suspension (OD_600_ = 1.0). Syringes were left undisturbed for 30 min, and the liquid inside the syringes was collected, serially diluted and plated on Petri-plates containing LBA medium. The plates were incubated overnight at 28°C. Following 24 h incubation bacterial colonies were counted as the average colony-forming units (CFU) obtained from 5 replicate-plates. The relative chemotactic response (RCR) was calculated, which represents the ratio of the CFU in response to the chemoattractant at a certain concentration to that of the control (sterile water). An RCR ratio > 2 is considered significant.

#### Method 2-Drop Assay

The drop assay (Yuan et al., 2015) was performed to trigger a chemotactic response by B26 bacterial cells. Briefly, B26 was grown in 50 mL of LB media with agitation at 160 rpm at 28°C. Pelleted cells were resuspended in 12 mL of sterile chemotaxis buffer (100 mM potassium phosphate [pH 7] with 20 μM EDTA) to which a 4 mL of 1% (v/v) of hydroxypropylcellulose solution was added. The cell suspension was placed in a 60 mm diameter Petri plate to which a 10 μL drop of 50 mM of each organic acid (succinic, fumaric, citric, oxalic, malic) or 50 x of concentrated root exudates collected from inoculated and control roots were added to the center of each Petri plate. Rings of turbidity that appeared in the next 30 min were recorded as an indication of the chemotactic response.

#### Method 3- Chemotactic Response of Strain B26 to Attractants in Carbon-Free Medium

This test was performed using the protocol of Kadouri et al. (2003). B26 was grown in LB as previously described. However, the cells were resuspended in potassium phosphate buffer (0.06 M, pH 6.8). Chemotaxis medium consisted of potassium phosphate buffer (pH 6.8), and 0.3% agar. 20 μL of above prepared B26 suspension was placed in the center of a 60 mm Petri plate containing chemotaxis media. Based on the capillary chemotaxis assay results, malic acid was selected as the attractant. Filter paper disks soaked in 50 μM concentration of malic acid were placed near the border of the plate equidistant from disks soaked in water as controls. The movement of bacterial cells toward malic acid was observed 24 h and 48 h post-inoculation. The experiment was replicated five times.

### Biofilm Quantification and Associated Traits

#### Biofilm Quantification Assay

To determine the effects of the root exudates and organic acids (OAs) on biofilm formation by strain B26, the biofilm assay was performed in 96-well microtiter plate as described by Yuan et al. (2015) with modification. B26 cells (OD_600_ of 1.0) were prepared as previously described. The bacterial suspension was pelleted by centrifugation at 8000 rpm for 10 min, washed twice with 1/2 MSgg medium (Branda et al., 2001), and resuspended in the same volume (5 mL) as the culture medium. To each well, 200 μL of 1/2 MSgg medium along with 10 μL of the above prepared bacterial suspension was added. Concentrated root exudates (50 x) or OAs were added to wells to obtain a final concentration of 10, 25, and 50 μM. The negative control consisted of culture medium alone. There were 6 replicates for each treatment. The plates were incubated for 24 h and 48 h at 37°C without shaking. Following incubation, the non-adherent cells were removed by washing with sterile distilled water, and the remaining adherent cells were stained with 200 μL of 0.1% solution of crystal violet. Plates were left undisturbed at room temperature for 30 min to allow proper staining of the biofilm cells. After 30 min, the excess crystal violet was removed by washing three times with distilled water, and plates were left to dry overnight. The crystal violet stain attached to the wells was later diluted by adding 200 μL of 4:1 (v:v) ethanol and acetone. Fifteen minutes later, solubilized crystal violet was transferred to a new microtiter plate and biofilm mass was quantified using a Synergy HT plate reader (Bio-TEK, Vermont, United States) at OD_570_.

#### Exopolysaccharides (EPS) Quantification Assay

EPS form the extracellular matrix of biofilm. To quantify EPS production by B26, the procedure of Krithiga and Jayachitra (2014) was adopted. Strain B26 was grown in 50 mL of sterile LB culture medium and incubated on a shaker incubator (120 rpm) at 28°C for 5 days. Following incubation, the bacterial cells were centrifuged at 10000 rpm for 20 min at 4°C. The resulting supernatant was filter sterilized using a 0.45 μm filter (Millipore filter, Ireland) to which 600 mL of previously chilled ethanol was added. The mixture was left overnight, undisturbed in the fridge to allow the precipitation of EPS. The supernatant was removed using a vacuum pump (Bio-Rad), and the EPS layer was collected by centrifugation, and its dry weight was recorded. The experiment was performed with six replications.

#### Alginate Quantification Assay

There is direct evidence that alginate functions to maintain cellular hydration, a function that has long been assumed and predicted but not demonstrated (Chang et al., 2007). To determine the amount of alginate produced by B26 under normal and hydric stress conditions, strain B26 was grown on LB and 5%; −0.47 MPA, PEG-amended LB broth, respectively. The isolation of alginate from the culture supernatant was performed according to the method of Knutson and Jeanes (1968). Cell-free supernatant was collected after centrifugation at 10000 rpm for 10 min. Alginate quantification was performed by measuring the uronic acid content from a standard curve of alginic acid of brown algae (Sigma Aldrich, United States), ranging from 10 to 1000 μg.mL^–1^ (May and Chakrabarty, 1994). Absorbance at A_530_ was indicative of a positive uronic acid test. The concentration of alginate production was measured in μg.mL^–1^ by comparing it with a standard curve. The experiment was performed with six replications for each treatment.

#### Hydrophobicity Assay

Microbial adhesion to hydrocarbons (MATH) assay was performed using the classical method of Rosenberg (2006). The bacterial suspension in LB was centrifuged at 8000 rpm for 10 min and the pellet was resuspended in phosphate magnesium buffer (pH 7.4). Three hundred μL of hydrocarbon, n-hexadecane (Alfa Aesar, United States) was added to the bacterial suspension, incubated for 10 min at 30°C, vortexed, and left undisturbed to allow for phase separation. The adherence of bacteria to the hydrocarbon was retrieved, and cell density absorbance was measured at 600 nm. The adhesion of bacteria to the hydrocarbon phase, *FPc* was calculated using the established formula (Zoueki et al., 2010): *FPC* = *(1-Af/A0) x100* where *Af* is the final absorbance after the addition of the hydrocarbon, *A0* is the original absorbance of bacterial cells before the addition of hydrocarbon. The experiment was performed with six biological replications.

#### Swimming and Swarming Motility Assays

Swimming and swarming motility assays were performed in LB Petri plates containing 0.3% (Swim plate) and 0.5% (Swarm plate) agar, respectively (Be’er and Harshey, 2011). Swarming motility but not swimming was tested in the presence of malic, citric, fumaric, succinic, and oxalic acids. Petri plates with LB and 0.5% agar were fortified with 10 μM of each organic acid. Each plate was inoculated with 3 μL of strain B26 (OD_600_ of 1) in the center and incubated for 24 h and 48 h at 28°C to determine the diameter of bacterial movement (mm). Assay plates were performed in six replicates.

### Scanning Electron Microscopy for Biofilm Formation on Glass Slide and Root Surface

#### Biofilm Formation *in vitro*

B26 was grown in LB medium as previously described. For imaging, 1 mL of the bacterial culture was placed on L-polylysine treated glass coverslips. Coverslips were incubated at 37°C for 48 h and were washed in 0.1 M phosphate buffer (pH 7.2). Biofilm formed on the coverslips was fixed overnight at 4°C in 4% formaldehyde solution (v/v) buffered with 0.1 M phosphate buffer (pH 7.2). Slides containing the biofilm-forming bacterium were dehydrated in an increasing series of ethanol (30–100%) with the last step repeated three times. This was followed by critical point drying of the slides using Leica EM-CPD300. The dried biofilm containing slides were coated with 4 nm of gold-palladium (Leica EM-ACE200) and examined using a Hitachi TM-1000 operating at 15 kV.

#### Biofilm Formation on Root Surface

A portion of the inoculated and control *Brachypodium* roots that were subjected before and after root exudate collection experiment were fixed overnight in 100% methanol following the procedure of Neinhuis and Edelmann (1996). Samples were subjected to constant slow shaking at room temperature, followed by three washes of 100% ethanol, 4 h each. Tissues were subjected to critical point drying (Leica EM-ACE200), and later coated with 4 nm gold-palladium and observed with a Hitachi TM-1000 operating at 15 kV. The sample preparation and image acquisition were performed at the McGill University Multi-Scale Imaging Facility, Sainte-Anne-de-Bellevue, Québec, Canada.

### Gene Expression Analysis

#### Expression Analysis of Organic Acid Genes in Wild Type *Brachypodium* Roots

To validate the observed trends of organic acids in root exudates and roots of *Brachypodium* wild type accession line Bd21-3, we examined the expression of genes encoding the respective organic acids in the TCA cycle (Table 2). Total RNA was extracted from flash-frozen pulverized 100 mg of root tissues 48 h post-inoculation with strain B26 and their respective controls using Spectrum^TM^ Plant Total RNA Kit (Sigma, Aldrich, United States) following the manufacturer’s protocols. RNA quality was confirmed on a denaturing formaldehyde agarose gel (1.2%) and quantified using a NanoDrop ND-100 spectrophotometer (Thermo Fisher Scientific). RNA (500 ng) was reverse-transcribed using One Script RT ABM kit (Vancouver, Canada) following the manufacturer’s protocols. Reverse transcription PCR assays were performed on 3 biological replicates and two technical replicates. Primer sets (Table 2) were designed based on sequences retrieved from Phytozome Bd21-3 v1.1 genome (Phytozome v12.1^6^). Primers were designed online from IDT website using Primer Quest Tool^7^. qRT-PCR conditions were optimized for each primer set and putative products were confirmed by sequencing. PCR amplification was performed in 10 μL reaction containing 1 x SYBR Green master mix (Bio-Rad,US), 200 nM of each primer, and 100 ng of cDNA template. The PCR thermal cycling parameters were, initial denaturation 95°C for 30 s followed by 40 cycles of 95°C for 15 s, annealing/extension 60°C for 30 s, along with dissociation curve at the end in Stratagene Mx3000 (Stratagene, Cedar Creek, United States). Transcript abundance was measured by using comparative threshold cycle (CT) method (2^–ΔΔCT^) (Livak and Schmittgen, 2001). Target genes were normalized over the housekeeping genes *ACTIN2*. BestKeeper tool^8^ was used to compare housekeeping genes *ACTIN2* and *UBC18*. *ACTIN2* had the lowest coefficient variation as compared to UBC18.

#### Gene Expression Analysis of T-DNA Mutant Lines

The relative transcript abundance of *ICDH* in *icdh* mutant line was compared with inoculated wild type Bd21-3 accession line. RNA isolation from roots, cDNA synthesis, and qRT-PCR reactions were carried out as described in the above section. Primer sets were designed for T-DNA mutant line JJ19999 based on sequences retrieved from Phytozome Bd21-3 v1.1 genome. (Phytozome v12.1^9^), and were checked for specificity to amplify only their target gene (Table 2). *ACTIN2* was used for normalization of relative transcript abundance levels.

#### Expression Analysis of Biofilm Associated Genes

The expression of biofilm-associated genes (*epsA, epsB, epsD, yqxM, bslA)* in response to various organic acids and root exudates were estimated by qRT-PCR. Cells of B26, obtained from the biofilm experiment, were induced in 1/2 MSgg medium amended with 25 μM concentration of each of the organic acids and 50x concentrated exudates released from inoculated and non-inoculated root after 24 h and 48 h as previously described in the biofilm quantification assay. Non-adherent cells were removed, and RNA was isolated only from adherent cells using NucleoSpin^®^ RNA isolation kit (Macherey-Nagel, Germany) based on the manufacturer’s protocol. Primer sets were designed from B26 genome and sequence information is present in Table 2. cDNA synthesis and qRT-PCR conditions were the same as described in the previous section. *RecA* was used as housekeeping gene to normalize data. Data were analyzed by 2^–Δ^ ^Δ^ ^*CT*^ method (Livak and Schmittgen, 2001).

### Statistical Analysis

Data of all experiments were analyzed using IBM Statistics SPSS Version 24 (SPSS Inc., Chicago, IL, United States). Comparison of mean was performed by Independent student *t*-test for comparison between control and inoculated samples. For analysis of more than two treatments as in the case of biofilm quantification and gene expression, data were analyzed by univariate analysis using the Tukey’s test (*P* ≤ 0.05) to determine the statistical significance of the treatments compared to their controls.

## Results

### Chemotactic Response of Strain B26 to Organic Acids

The chemotaxis capillary assay revealed that strain B26 was attracted to a variety of organic acids and that B26 cell migration was positively linked to organic acid concentrations (Table 3). Significant (*P* < 0.05) higher numbers of cells were induced by fumaric and citric acids at concentration of 25 μmol.L^–1^, followed by 10 μmol.L^–1^ of oxalic acid and 50 μmol.L^–1^ of malic acid. The chemotactic response of B26 to oxalic acid at concentrations higher than 10 μmol.L^–1^, and citric acid at concentrations of 10 and 50 μmol.L^–1^ were similar to those in sterile water (control), and did not induce a chemotactic response (Table 3). RCR, the ratio of cell number attracted to organic acids relative to control was significantly (*P* ≤ 0.05) high with malic acid (17.6) at 50 μmol.L^–1^, and citric acid (7.5) at 25 μmol.L^–1^. This indicates that a significant induction of chemotactic response with increasing concentrations of malic acid (Table 3).

**TABLE 3:** Chemotaxis of a B26 toward different organic acids.

Chemoattractant	Chemoattractant concentration (μmol.L^–^^1^)	CFU.mL^–^^1^*	RCR
Malic acid	Control	0.36 ± 0.02^c^	
	10	4.31 ± 0.26^b^	11.87
	25	5.10 ± 0.44^b^	14.07
	50	6.37 ± 0.26^a^	17.59
Citric acid	Control	1.05 ± 0.41^b^	
	10	2.22 ± 0.37^bc^	2.11
	25	7.86 ± 0.22^a^	7.45
	50	0.64 ± 0.11^b^	0.62
Fumaric acid	Control	5.12 ± 0.65^c^	
	10	0.55 ± 0.05^b^	0.11
	25	13.56 ± 0.37^a^	2.65
	50	0.51 ± 0.06^b^	0.1
Succinic acid	Control	0.36 ± 0.02^c^	
	10	1.21 ± 0.14^b^	3.31
	25	0.94 ± 0.06^b^	2.61
	50	2.16 ± 0.22^a^	5.95
Oxalic acid	Control	2.76 ± 1.35^b^	
	10	7.35 ± 0.52^a^	2.65
	25	3.29 ± 0.39^b^	1.18
	50	3.15 ± 0.23^b^	1.14

* CFU numbers in columns represent the means of CFU × 10^3^ ± standard error of the mean × 10^3^ of 4 replicates. Means of specific chemoattractant with different letters within a column differ significantly according to the Tukey’s test (P ≤ 0.05). RCR, relative chemotactic ratio is calculated based on the ratio of the colony-forming units (CFU × 10^3^) in response to the chemoattractant to the CFU × 10^3^ of the control (sterile water). An RCR ratio > 2 is considered significant.

### Motility and Chemotaxis-Associated Traits of *Bacillus velezensis* Strain B26 Were Triggered by Selected Organic Acids

**FIGURE 1:**
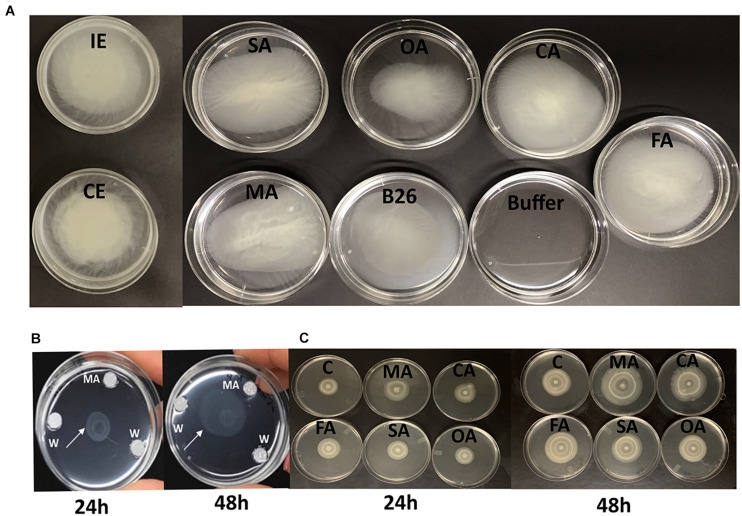
**(A)** Chemotactic response of *Bacillus velezensis* B26 toward different organic acids, inoculated (IE), and non-inoculated concentrated root exudates (CE) of Bd21-3. **(B)** Chemotactic responses of B26 toward malic acid (MA) after 24 h and 48 h. **(C)** Bull’s eye type swarming pattern made by B26 on 0.5% agar fortified with organic acids after 24 h and 48 h. MA, malic acid; CA, citric acid; FA, fumaric acid, SA, succinic acid, OA, oxalic acid; C, water.

All tested OAs (succinic, oxalic, citric, malic, and fumaric acids), and concentrated root exudates initiated a chemotactic response on B26 cells compared to the bacteria alone and buffer solution (Figure 1A). All organic acids, except oxalic acid induced relatively large rings of turbidity. Compared to organic acids, root exudates collected from inoculated (IE) and non-inoculated (CE) *Brachypodium* plants triggered an intense chemotactic response within 30 min with dense turbidity pattern, indicating that root exudates actively recruit cells of B26 (Figure 1). Based on the high RCR ratio of malic acid with increasing concentrations (Table 3), the motility of strain B26 to malic acid as an attractant was illustrated in Figure 1B. The motility cells in the form of a turbid band of B26 cells were visible at 24 h and expanded in the direction of the malic acid, after 48 h (Figure 1B) as compared to disks imbibed with water (control).

### Biofilm Associated Traits

Biofilm associated traits of strain B26 including quantification of EPS, alginate, hydrophobicity and swarming motility were determined. As shown in Table 4, the production of EPS by B26 was 868 μg.mL^–1^. The production of alginate with hydric stress treatment led to an increase of (18%) compared to media without PEG. The percent hydrophobicity of B26 (69%) was quantified as the fraction of bacteria adhered to the hydrocarbon phase.

**TABLE 4:** Biofilm-associated traits and swarming and swimming motility of B26.

Biofilm Characteristics^#^			Swimming motility (zone diameter mm)^$^		Swarming motility (Swarm diameter mm)*	
			
		Treatment^#^	24 h	48 h	24 h	48 h
Hydrophobicity (%)	69	Control	10.7 ± 0.3^b^	30.54 ± 1.5^a^	25.3 ± 0.3^b^	40.0 ± 2.8^a^
EPS (μg.mL^–1^)	868.3 ± 22.0	Malic acid	NA	NA	31.0 ± 2.0^a^	48.3 ± 3.3^a^
LB + Alginate (μg.mL^–1^)	375.2 ± 17.9^a^	Citric acid	NA	NA	28.0 ± 1.1^b^	43.3 ± 4.4^a^
5%PEG + Alginate (μg.mL^–1^)	456.0 ± 24.0^b^	Fumaric acid	NA	NA	27.6 ± 1.4^b^	39.0 ± 2.0^a^
		Succinic acid	NA	NA	24.6 ± 0.8^b^	36.6 ± 3.3^a^
		Oxalic acid	NA	NA	24.0 ± 1.1^b^	38.6 ± 3.1^a^

^#^Hydrophobicity, EPS, Exopolysaccharide, alginate, swimming and swarming motility represent the average of six replicates. ^$^Means within rows with different superscript letters are significant according to Independent t-Test (P ≤ 0.05). NA, not applicable.*Means within columns with different superscript letters are significant according to according to Tukey’s test (p ≤ 0.05).

To investigate whether strain B26 has the ability of swimming and swarming, B26 was grown on “Swim plates” fortified with 0.3% and Swarm plates fortified with 0.5% agar. After 48 h of incubation, an extensive and significant (*P* ≤ 0.05) increase in the diameter of the swimming zone traveled by bacteria in the Swim plate was observed (Table 4). The diameter of swarm significantly increased exhibiting the Bull’s eye swarming phenotype in plates fortified with malic acid at 24 h (Figure 1C and Table 4). Compared to the control, other organic acids did not affect swarming motility. After 48 h, there were no differences between the control and the swarming patterns of B26 in plates fortified with organic acids.

### Exogenously-Added Organic Acids and Root Exudates Enhanced Biofilm Formation in Strain B26

The biofilm formation of strain B26 in response to organic acids and root exudates was measured using a quantitative microtiter plate assay at 24 h and 48 h incubation. Irrespective of the type of organic acid, concentration and time of incubation, the biofilm production of B26 significantly (*P* ≤ 0.05) increased after 24 and 48 as compared to the control (Table 5). The relative fold increase (RFI) values of biofilm formation of B26 cells to culture media in response to organic acids at 24 h ranged from 2.4 for citric acid (25 and 50 μM concentrations) to 3.8 for fumaric acid (10 μM concentration). Whereas the RFI values of biofilm formation at 48 h incubation ranged from 3 for succinic acid (10 μM concentration) to 9.2 for citric acid (50 μM concentration). The effect of *Brachypodium* exudates collected from non-inoculated and inoculated roots on biofilm formation significantly increased relative to the control after incubating the microtiter plates for 24 h and 48 h. The RFI values of both treatments relative to the control at 24 h was 5.8 and 7.7, and at 48 h RFI values were 5.7 and 7.7, respectively. However, induction of biofilm formation in response to root exudates sampled from inoculated and non-inoculated roots was similar (Table 5).

**TABLE 5:** Effect of different concentrations of organic acids and concentrated root exudate of *Brachypodium* accession Bd21-3 on biofilm formation of *Bacillus velezensis* B26 in 1/2 MSgg medium.

Treatment*	Concentration	Biofilm formation (OD_570_, 24 h)	RFI(OD_570_,24 h)	Biofilm formation (OD_570_, 48 h)	RFI (OD_570_, 48 h)
Malic acid	Media $	0.14 ± 0.01^b^	–	0.16 ± 0.01^b^	–
	10 μM	0.50 ± 0.01^a^	3.6	0.55 ± 0.04^a^	3.4
	25 μM	0.46 ± 0.02^a^	3.3	0.53 ± 0.02^a^	3.3
	50 μM	0.46 ± 0.01^a^	3.3	0.54 ± 0.02^a^	3.4
Citric acid	Media	0.14 ± 0.01^b^	–	0.16 ± 0.01^b^	–
	10 μM	0.42 ± 0.01^a^	3	0.56 ± 0.01^a^	3.5
	25 μM	0.34 ± 0.01^a^	2.4	1.16 ± 0.14^a^	7.3
	50 μM	0.33 ± 0.02^a^	2.4	1.47 ± 0.20^a^	9.2
Fumaric acid	Media	0.14 ± 0.01^b^	–	0.16 ± 0.01^b^	–
	10 μM	0.53 ± 0.01^a^	3.8	0.55 ± 0.02^a^	3.4
	25 μM	0.51 ± 0.03^a^	3.6	0.52 ± 0.01^a^	3.3
	50 μM	0.45 ± 0.02^a^	3.2	0.51 ± 0.01^a^	3.2
Oxalic acid	Media	0.14 ± 0.01^b^	–	0.16 ± 0.01^b^	–
	10 μM	0.38 ± 0.01^a^	2.7	0.49 ± 0.02^a^	3.1
	25 μM	0.39 ± 0.02^a^	2.8	0.97 ± 0.07^a^	6.1
	50 μM	0.37 ± 0.01^a^	2.6	1.22 ± 0.09^a^	7.6
Succinic acid	Media	0.14 ± 0.01^b^	–	0.16 ± 0.01^b^	–
	10 μM	0.41 ± 0.02^a^	2.9	0.48 ± 0.03^a^	3
	25 μM	0.38 ± 0.04^a^	2.7	0.52 ± 0.03^a^	3.3
	50 μM	0.41 ± 0.02^a^	2.9	0.54 ± 0.04^a^	3.4
Root exudate	Media	0.14 ± 0.01^b^	–	0.16 ± 0.01^b^	–
	Root exudates B −	0.81 ± 0.04^a^	5.8	0.91 ± 0.06^a^	5.7
	Root exudates B +	1.08 ± 0.07^a^	7.7	1.23 ± 0.05^a^	7.7

* Numbers represent the mean of 5 replicates ± standard error of the mean. Superscript letters within a column represent significance according to Tukey’s test (P ≤ 0.05). ^$^1/2 MSgg is the medium specific for biofilm growth of Bacillus; Root exudates B-, Non-inoculated roots; Root exudates B+, Inoculated with B26; RFI, Relative Fold Increase, RFI is ratio of the treatment to media.

### Strain B26 Forms Biofilm on Abiotic Surfaces and *Brachypodium* Root Surfaces

**FIGURE 2:**
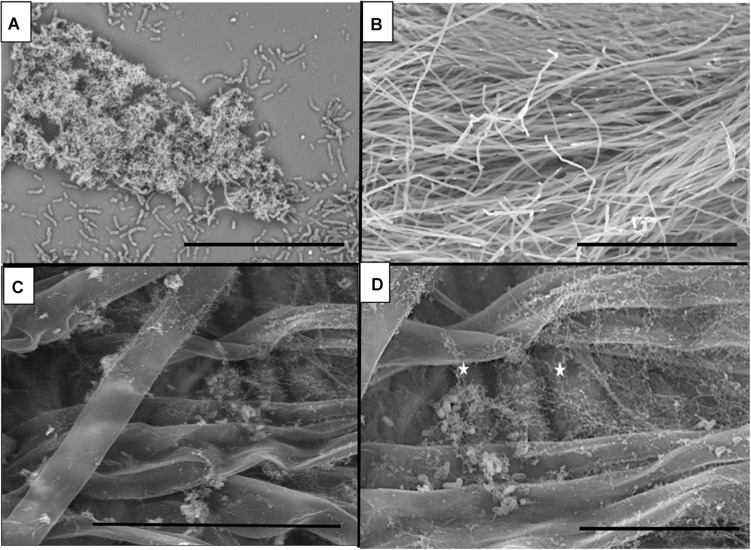
Scanning electron microscopy (SEM) micrographs of **(A)** Biofilm formation by B26 on glass surface (Bar = 30μm). **(B)** Control *Brachypodium distachyon* Bd21-3 roots (Bar = 300 μm). **(C)** Biofilm formation by B26 on the surface of roots prior to root exudate collection of Bd21-3 (Bar = 50μm). **(D)** Magnified view of biofilm formed by B26 on Bd21-3 roots after root exudate collection (Bar = 20 μm).

Bacterial cells of B26 were visualized for biofilm formation on glass coverslips and root surfaces of *Brachypodium.* Formation and adherence of B26 biofilm was observed on glass slides (Figure 2A). On *Brachypodium* roots aggregates of B26 rod-shaped cells were encased in a network of mucilage surrounding the roots before and after root exudate collection (Figures 2C,D). No such network was observed in control non-inoculated *Brachypodium* roots (Figure 2B). Additionally, the presence of strain B26 was confirmed on roots prior and after root exudate collection using specific primer set for B26 (Forward Primer 5′CAAGTGCCGTTCAAATAG3′, Reverse Primer 5′CTCTAGGATTGTCAGAGG 3′) (Supplementary Figure S2).

### Strain B26 Modulated the Levels of Organic Acids in Root Exudates and Roots of *B. distachyon*

**FIGURE 3:**
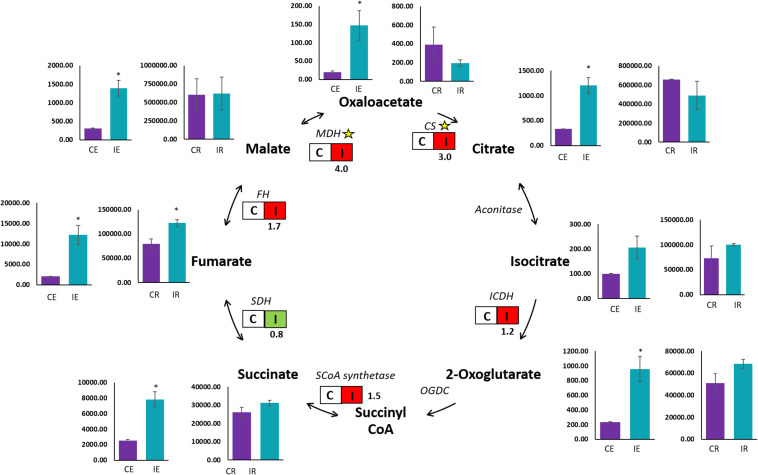
GC-MS and transcript analysis of TCA cycle genes in Bd21-3 roots inoculated with B26 for 48 h. Bar graphs represent the GC-MS results of control roots (CR), inoculated roots (IR), control root exudates (CE), and inoculated root exudates (IE). Significant changes are shown by an *asterisk* according to Independent *t*-Test (*P* ≤ 0.05). Transcription of encoding genes of the TCA cycle are shown by boxes with non-inoculated roots (C) and inoculated roots (I). Red illustrates an increase in relative transcript abundance in response to B26, and green represents a decrease in expression. Fold change in transcript abundance is indicated below each box. Yellow stars represent significant differences (*P* ≤ 0.05) between control and treatment. CS, Citrate synthase; ICDH, Isocitrate dehydrogenase; SCoA synthetase, Succinyl CoA synthetase; SDH, Succinate dehydrogenase; FH, Fumarate hydratase; MDH, Malate dehydrogenase.

A qualitative GC-MS analysis of organic acids in the TCA cycle was performed on roots and root exudates of *Brachypodium* along with their respective controls. Chemical compounds were identified by peak data on the chromatogram (Supplementary Figure S3). Peak areas observed as the quantifier ions of the TCA cycle metabolites were for succinic, fumaric, oxaloacetic, malic, 2-ketoglutaric, aconitic, citric, isocitric, pyruvic and lactic acids. Organic acids in root exudates were measured in terms of relative peak area.mL^–1^, while organic acids in *Brachypodium* roots were measured by relative peak area.mg^–1^. A significant (*P* ≤ 0.05) increase was observed in oxaloacetic, malic, fumaric, citric, succinic and 2-ketoglutaric acids in root exudates from B26 inoculated *Brachypodium* roots (IE), compared to root exudates (CE) of the control (Figure 3). The relative peak area of fumaric acid in exudates of inoculated roots (IE) was the highest among all the organic acids. In contrast, in the case of roots, the maximum peak area was observed in the malic and citric acids irrespective of treatment or control, indicating the highest production of these two organic acids in roots. However, only fumaric acid significantly increased in inoculated roots (IR) as compared to control roots (CR), while the remaining organic acids in inoculated roots (IR) had similar levels as their respective controls (Figure 3).

### Strain B26 Triggered the Regulation of Organic Acid Genes Encoding the TCA Cycle

To validate the observed trends of organic acids in roots, we examined transcript abundance of the following organic acid genes [*Citrate synthase* (*CS*), *Isocitrate dehydrogenase* (*ICDH*), *Succinyl-CoA synthetase*, *Succinate dehydrogenase* (*SDH*), *Fumarate hydratase* (*FH*) and *Malate dehydrogenase* (*MDH*)] in tissues of inoculated and control roots by qRT-PCR. A significant upregulation in transcripts abundance of *CS* and *MDH* by 3-fold (*P* = 0.005) and 4-fold (*P* = 0.002), respectively were observed in tissue of the inoculated roots (I) (Figure 3). However, a slight increase (*P* >0.05) in transcript abundance of *FH* (1.7-fold), *ICDH* (1.2-fold) and *Succinyl-CoA synthetase* (1.5-fold) was observed in inoculated roots. On the other hand, transcripts of *Succinate dehydrogenase* (*SDH*) were downregulated in inoculated roots relative to the control (Figure 3).

### Gene Expression Analysis of *icdh* Mutant lines

**FIGURE 4:**
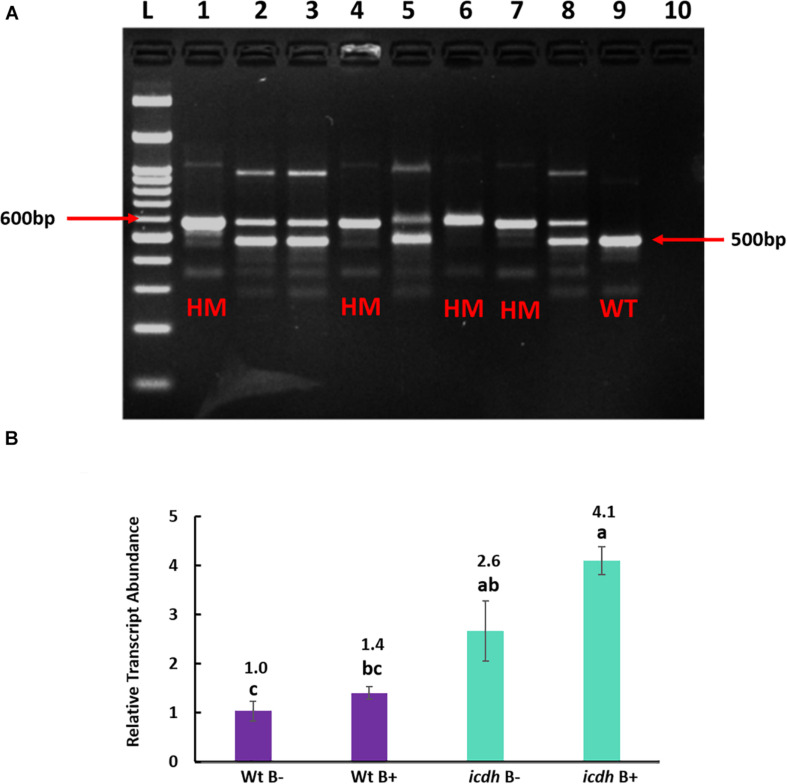
**(A)**. Genotyping of homozygous (HM) *Isocitrate dehydrogenase* (*icdh*) mutants of Bd21-3. L: 100 bp ladder, lanes 1–8: DNA from different *Isocitrate dehydrogenase* of Bd21-3 T-DNA line JJ19999, lane 9: Wild Type Bd21-3 (WT), lane 10: Negative Control. **(B)** Relative transcript abundance of gene *ICDH* in non-inoculated wild type Bd21-3 roots (WtB–); inoculated wild type Bd21-3 roots (WtB+); selected homozygous mutant (*icdh* B–) and inoculated mutant (*icdh* B+). Numbers above the bar graphs represent significant fold change relative to the control (WtB–). Means with same letters are not significant, while means with different letters are significant according to Tukey’s test (*p* ≤ 0.05).

Multiplex PCR of T-DNA mutants for genes *Succinate dehydrogenase*, *Malate dehydrogenase* and *Citrate synthase* produced heterozygous bands even after two generations. Only lines of mutant NADP-dependent *Isocitrate dehydrogenase(icdh)* produced single homozygous bands of 600 bp compared to the wild type accession line Bd21-3 with a band size of 500 bp, indicating the absence of an insert (Figure 4A). Hence, only homozygous T-DNA *icdh* lines were retained for expression analysis. As expected, the transcript abundance of gene *ICDH* in non-inoculated mutant *icdh* line was significantly (*P* = 0.001; 2.6-fold) higher than in WtB- (Figure 4B). In response to B26 inoculation, a 4.1-fold (*P* = 0.045) in the mutant *icdh*B + compared to the WtB- was observed, (Figure 4B). There was no difference in transcript abundance of *ICDH* between WtB- and WtB + . Nevertheless, a slight increase (1.6-fold), although not statistically significant, in transcript abundance of *icdh*B + relative to *icdh*B- was observed.

### Organic Acids Induced Significant Changes in Biofilm Formation Through Activation of Genes Related to Biofilm and Genes Related to Hydrophobin in Strain B26

**FIGURE 5:**
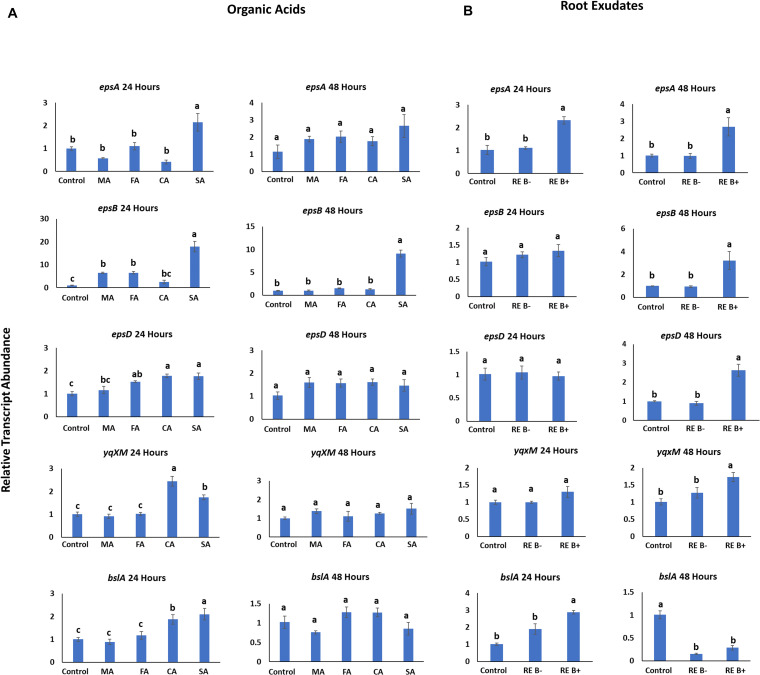
Transcript abundance of biofilm-related genes in induced B26 biofilm after 24 h and 48 h of induction with **(A)** various organic acids. MA, malic acid, FA, fumaric acid, CA, citric acid, SA, succinic acid. **(B)** with exudates from non-inoculated (RE B-) and inoculated roots (RE B +) and control (without root exudates). Letters (a,b,c) above the bar graphs represent means with significance according to Tukey’s test (*P* ≤ 0.05).

Transcript abundance of biofilm genes related to EPS formation; *epsA, epsB, epsD*; the gene *yqxM* encoding for membrane protein and *bslA* which encodes the hydrophobin component of the biofilm, were induced by specific organic acids. Organic acids caused a differential response of genes related to EPS formation at 24 h compared to the control (Figure 5A). The genes, *epsA*, and *epsB* were significantly upregulated by succinic acid, with a fold increase of 2.1 (*P* = 0.012), and 17.9 (*P* = 0.001), respectively, when compared to the control and other organic acids. Fumaric and malic acids significantly increased the transcription of *epsB* with 6.5-fold increase (*P* = 0.033) and 6.4-fold increase (*P* = 0.036), respectively. The gene *epsD* was also upregulated by succinic and citric acids (1.8-fold; *P* = 0.004), and fumaric acid (1.5-fold; *P* = 0.04) when compared to the control. However, there was no difference in *epsD* transcript abundance by malic when compared to the control. Genes *yqxM* and *bslA* in strain B26 were significantly induced by citric and succinic acids compared to other organic acids and control at 24 h. On the other hand, after 48 h of biofilm induction, transcript abundance of all the genes was almost similar in every treatment relative to the control. In contrast, succinic acid induced a 9.1-fold increase (*P* = 0.001) in transcript abundance of *epsB* (Figure 5A).

### *Brachypodium* Root Exudates Differentially and Temporally Stimulated Biofilm-Related Genes

The impact of root exudates on biofilm genes was also measured after 24 h and 48 h post-inoculation. At 24 h post-inoculation, transcript abundance of biofilm and extracellular matrix production genes were not significantly altered compared to control, except for *epsA* (*P* = 0.002); and the self-assembly hydrophobin encoding gene *bslA* (*P* = 0.04) that coats the biofilm, (Figure 5B). While at 48 h post-inoculation, genes encoding the EPS biofilm formation and *yqxM* encoding gene for membrane protein were significantly and temporally transcribed in response to exudates of inoculated roots. The highest fold increase was observed in *epsA* and *epsB* (3.2-fold) followed by *epsD* (2.6-fold) compared to exudates of non-inoculated roots and control (Figure 5B). Interestingly, significant downregulation of *bslA* was observed relative to the control at 48 h post-inoculation.

## Discussion

We previously reported on the successful colonization of *Brachypodium distachyon* Bd21 by the plant growth-promoting bacterium (PGPR), *B. velezensis* strain B26. Strain B26 effectively increased root and shoot weights, and accelerated growth rate and seed yield when plants were grown in a potting mix (Gagné-Bourque et al., 2015). In this study, we focused on the relation between *B. velezensis* strain B26 growth, chemotaxis, biofilm formation, and the role of *Brachypodium* root exudates in promoting colonization.

It is now recognized that successful colonization by PGPR and endophytes involves the initiation of cross-talk of signal molecules that originate from root exudates (De Weert et al., 2002; Badri and Vivanco, 2009; Cao et al., 2011; Feng et al., 2018), and elicit a chemotactic response by bacterial endophytes (Yaryura et al., 2008; Yuan et al., 2015). Root exudates include diverse groups of metabolites ranging from simple organic anions to complex polymer mucilages (Jones, 1998), and are considered a triggering factor for bacterial chemotaxis. Some of the components may serve as positive attractants leading to the recruitment and colonization of beneficial bacteria or as negative attractants to repel pathogens and parasitic plants (Badri and Vivanco, 2009; Liu et al., 2017; Feng et al., 2018). Simple organic anions including lactate, oxalate, succinate, fumarate, malate and citrate being the primary anion components of root exudates, some of which are vital signals to specifically induce directed motility and chemotactic response of beneficial soil bacteria (Rudrappa et al., 2008; Canarini et al., 2019).

Therefore, a semi-hydroponic system for the collection of roots exudates from intact *Brachypodium* root system grown under sterile conditions was developed using glass beads as an inert substrate to support root growth. This system facilitated the collection of sterile root exudates and the metabolite profiling of organic anions from intact root systems. The use of inert substrates such as glass beads or sand instead of soil or clay that strongly absorbs a variety of metabolites may affect exudation and metabolite profiles. Notably, root exudation studies of *Brachyodium* grown under sterile hydroponic systems resulted in similar metabolite profiles of exudates in sand or glass beads (Kawasaki et al., 2016; Sasse et al., 2019). The most common organic acids released from *Brachypodium* roots were malate, citrate, succinic, fumarate, and oxalate (Kawasaki et al., 2016). In this study, roots and root exudates of *Brachypodium* had similar composition of organic acids. However, fumarate and succinate were the most abundant organic acids followed by malate, citrate and isocitrate released from exudates of *Brachypodium* at 48 h post-inoculation with strain B26 compared to those released from control root exudates. Root excretions of selective and significant amounts of fumarate and succinate followed by malate and citrate may indicate that these organic acids act as chemoattractants for strain B26 and may play an important role in root colonization of *Brachypodium* (Badri and Vivanco, 2009). In parallel, we also validated the observed trends of organic acids in roots by examining the transcription abundance of genes encoding the respective organic acids in TCA cycle. Malate and citrate are intermediate metabolites of the TCA cycle, and their accumulation in root cells may result in their transcription and synthesis. As expected, a significant upregulation of genes encoding *MDH* and *CS* was observed in inoculated roots (Figure 3). In contrast, B26 did not trigger a significant transcript induction of *ICDH* in inoculated roots (I) relative to the control (C). Accordingly, B26 inoculation of *icdh* mutant line did not affect both the *icdh* mutant and the wild type.

Given the unequal distribution of solutes, including organic acids in roots and root exudates, we did not attempt to correlate the internal concentrations to root exudation of organic acids with the assumption that higher internal concentration would lead to higher root exudation is misleading (Mariano et al., 2005). Certain components of root exudates, including organic acids (e.g., fumarate, citrate, malate, and succinate) can positively trigger the induction of genes involved in the matrix and biofilm formation (Rudrappa et al., 2008; Zhang et al., 2014; Yuan et al., 2015). In this study, the establishment that fumarate, succinate, malate and citrate were the most abundant organic acids in root exudates of inoculated *Brachypodium* roots, prompted us to study the transcription of genes encoding the components of the extracellular matrix of the biofilm, *epsA,B,D; yqxM* involved in biofilm matrix formation (Branda et al., 2006) and *bslA* for self-assembling the hydrophobin that coats the biofilm (Zhang et al., 2015). Succinic acid distinctly and temporally triggered the upregulation of *epsA,epsB* genes encoding biofilm. While citric acid upregulated *yqxM* that is required for biofilm formation, and *bslA.* Fumaric acid and malic acid induced *epsB*. Similarly, *Brachypodium* root exudates also positively influenced the biofilm formation gene *epsA* in strain B26. These findings agree with Zhang et al. (2015). They showed that root exudates and individual organic acid viz., fumaric, citric, malic, and succinic acids can trigger the differential induction of several biofilms and matrix formation genes of *Bacillus amyloliquefaciens* SQR9 in maize roots. Our findings suggest that selective organic acids and root exudates of *Brachypodium* are involved in biofilm induction.

The down regulation of the *bslA* gene at 48 h but not at 24 h in response to root exudates may well be due to the age of biofilm cells. The arrested growth of *Bacillus* biofilm after 50 h of incubation terminated the synthesis of biofilm matrix components (i.e., exopolysaccharides and the hydrophobin protein BslA) (Bartiloni et al., 2019). The reduction of *bslA* gene expression by root exudates after 48 h of incubation, may be to the onset of biofilm cells’ age. The secreted protein, BslA is an essential component of biofilm surface repellency of *B. subtilis* biofilms. Once the surface repellency is lost, the biofilm starts disrupting (Kobayashi and Iwano, 2012).

For successful root colonization, chemotaxis and biofilm formation are the two most crucial activities performed by PGPR (Van de Broek et al., 1998; Zhang et al., 2014; Bhattacharyya et al., 2017). Bacterial motility has an essential role in biofilm formation (Houry et al., 2010; Allard-Massicotte et al., 2016). In our study, the chemotactic response toward concentrated root exudates of *Brachypodium*, and a variety of individual organic acids that are involved in the TCA cycle played a functional role as signaling molecules, and initiated a chemotactic response using the quantitative capillary assay. A strong chemotactic response of B26 was observed particularly toward malic acid, followed by citric acid, succinic acid, and fumaric acid. Equally, under conditions of carbon-free medium, the motility of strain B26 had almost doubled in the presence of malic acid. These findings indicate that malic acid sustained bacterial growth in the absence of any other external compounds and reinforced the notion that organic acids in root exudates, and individual organic acids, can initiate a chemotactic response in strain B26 leading to biofilm formation. All organic acids, except oxalic acid, were capable of inducing a strong chemotactic response. These results agree with recent studies showing organic acids released from the roots of banana and tomato help the colonization *of Bacillus amyloliquefaciens* NJN-6 and *B*. *amyloliquefaciens* T-5, respectively (Tan et al., 2013; Yuan et al., 2015). Consistent with our results, the above studies also showed that oxalic acid did not induce a strong chemotactic response of their bacteria. Moreover, concentrated *Brachypodium* root exudates initiated strong chemotactic response. However, we cannot ignore that signaling compounds other than organic acids, including sugar, amino acids, and phenolic compounds are important components of the plant root exudates and could also serve as signals (Badri and Vivanco, 2009). In summary, the ability of B26 cells to move toward *Brachypodium* roots in response to carbon-containing compounds and proliferate is an essential trait that enables strain B26 to be competitive in the rhizosphere.

The enhancement of biofilm formation in response to root exudates was previously reported in *Bacillus velezensis* strain S3-1 in maize (Jin et al., 2019), *Bacillus velezensis* strain FZB42 in tomato (Al-Ali et al., 2018), *B. subtilis* in *Arabidopsis* and tomato (Rudrappa et al., 2008; Chen et al., 2012) and *Bacillus amyloliquefaciens* NJN-6 in banana root exudates (Yuan et al., 2015). To further confirm that root exudates and exogenously-added organic acids affect biofilm formation of strain B26, quantitative microtiter assay showed that citric acid and oxalic acid promoted maximum biofilm formation after 48 h compared to the control, and most importantly, *Brachypodium* root exudate stimulated biofilm formation of strain B26. Similar results were reported where citric acid stimulated biofilm formation by *Bacillus amyloliquefaciens* SQR9 (Zhang et al., 2014) and oxalic acid induced biofilm formation by *Bacillus velezensis* Strain S3-1 (Jin et al., 2019).

Bacterial biofilms are congregations of bacterial cells within a matrix composed of EPS, alginates, and some proteins that contribute to the adherence of root systems (Davey and O’toole, 2000; Czaczyk and Myszka, 2007). Plant growth-promoting bacteria can take advantage of nutrients from root exudates to reproduce and facilitate the biofilm formation. The adhesion of bacteria to a solid surface in the form of biofilm is influenced by various traits viz; bacterial cell surface hydrophobicity; EPS production, and swarming ability (Nagórska et al., 2010; Al-Abbasi, 2018). PGPR most effectively mitigate the impact of abiotic stresses on plants through the production of polysaccharides and biofilm. Interestingly, our strain B26 exhibited all of the biofilm mentioned-associated traits. The bacterial polysaccharide and alginate play a vital role in maintaining the biofilm architecture and providing stress tolerance to plants (Halverson, 2009). Direct evidence points to the function of alginate in maintaining cellular hydration and biofilm formation under desiccation conditions (Chang et al., 2007). Our study showed evidence that when strain B26 is under hydric stress, alginate production is substantially increased by 18%. These results may implicate the role of alginate in desiccation tolerance of B26 (Gagné-Bourque et al., 2015). Previously, we reported that inoculation of *Brachypodium* and timothy grass with B26 affected the whole growth cycle of the plants, by accelerating the growth rates, lessening drought stress after 8 weeks, and improving plant growth through the osmolyte accumulation in roots and shoots (Gagné-Bourque et al., 2015; Gagné-Bourque et al., 2016). The notion that alginate also contributes to maintaining a hydrated microenvironment protecting B26 residents from desiccation and facilitating biofilm formation under stressful conditions may well be entertained and requires further testing.

Inoculation of *Brachypodium* roots with strain B26 allowed us to visualize the biofilm formation on roots prior and after collection of root exudates and compare it to that developed on the abiotic surface (glass slides). As expected, different structures of the matrix were detected on the abiotic and biotic surfaces. A mesh-like form adhered to inoculated *Brachypodium* roots before and after root exudate collection. Hence, rinsing the roots in ultrapure water did not affect the biofilm formation on inoculated roots and is required for cell fixation and colonization of plant tissues (Gagné-Bourque et al., 2015; Posada et al., 2018). This matrix was absent in control roots. The biofilm has advantages to bacterial cells because it gives them protection from predators, provides a physical barrier against the diffusion of unwanted molecules and helps them retain nutrients (Bogino et al., 2013). Similar types of biofilm matrix were formed by *Bacillus subtilis* EA-CB0575 in tomato and banana roots (Posada et al., 2018).

## Conclusion

In conclusion, our results indicate that colonized roots by PGPR improve the composition of root exudate and facilitate the chemotaxis and biofilm formation. We hypothesize that the biofilm induction by OAs is due to the upregulation of various biofilm-associated genes. Moreover, chemotaxis and biofilm formation results indicate that the strain B26 is primed for the endophytic lifestyle by organic acid and root exudates. These findings increased our understanding of molecular mechanisms behind the role of organic acid and root exudates in recruiting PGPR. Our results imply that strain B26 is involved in modulating the organic acids of the TCA cycle. The increase in the transcription of TCA cycle intermediates in inoculated *Brachypodium* roots indicates the role of B26 in improving root exudate composition. This is the first report describing the effect of PGPR on TCA cycle genes in plants. Yet our current knowledge on the quantitative composition of organic acids and other compounds excreted by *Brachypodium* roots is still fragmented, and these further merit studies.

## Data Availability Statement

The raw data supporting the conclusions of this article will be made available by the authors, without undue reservation.

## Author Contributions

MS and SJ: conception and design of the study and interpretation of data for the work. MS and DS: acquisition of data for the study. MS: analysis of data for the work. MS, DS, J-BC, and SJ: manuscript revision and approval. All authors contributed to the article and approved the submitted version.

## Conflict of Interest

The authors declare that the research was conducted in the absence of any commercial or financial relationships that could be construed as a potential conflict of interest.

## Footnotes

1 https://jgi.doe.gov/our-science/science-programs/plant-genomics/brachypodium/brachypodium-t-dna-collection/

2 http://1ofdmq2n8tc36m6i46scovo2e-wpengine.netdna-ssl.com/wp-content/uploads/2015/05/T-DNA-genotyping.pdf

3 https://phytozome.jgi.doe.gov/pz/portal.html

4 http://1ofdmq2n8tc36m6i46scovo2e-wpengine.netdna-ssl.com/wp-content/uploads/2015/05/T-DNA-genotyping.pdf

5 https://jgi.doe.gov/our-science/science-programs/plant-genomics/brachypodium/brachypodium-t-dna-collection/

6 https://phytozome.jgi.doe.gov/pz/portal.html

7 https://www.idtdna.com/PrimerQuest/Home/Index

8 https://www.gene-quantification.de/bestkeeper.html

9 https://phytozome.jgi.doe.gov/pz/portal.html

## Supplementary Material

Supplementary Figure 1 **(A)** Semi-Hydroponics system in Magenta GA-7 tissue culture boxes for growing Bd21-3 under sterile conditions in glass beads saturated with 1/4th Hoagland’s solution. **(B)** Growth of roots in magenta boxes. **(C)** Root exudate collection system.

Supplementary Figure 2 PCR amplified products on 1% Agarose gel using specific B26 primers. L, 3Kb ladder**;** lane1, amplified DNA of Bd21-3 from inoculated *Brachypodium* roots before exudate collection. lane 2, amplified DNA product of Bd21-3 from inoculated *Brachypodium* roots after exudate collection. Lane 3; amplified DNA from culture of B26. Lane 4: negative control.

Supplementary Figure 3 Gas Chromatograms acquired in Scan mode for Bd21-3 samples: **(A)** Root Extracts Control **(B)** Root Extract Inoculated **(C)** Roots Control **(D)** Roots Inoculated 1, Carbonate (always present, even in blanks); 2, Lactic acid; 3, Boric; 4, Succinic; 5, Fumaric; 6, Phosphoric; 7, 2-ketoglutaric; 8, Myristic-d27 (Internal standard); 9, Malic; 10, Aconitic; 11, Dopamine; 12, Citric; 13, Isocitric.
