# Supplementary figures and images for: A Crosstalk Between Brachypodium Root Exudates, Organic Acids, and Bacillus velezensis B26, a Growth Promoting Bacterium
Source: Front Microbiol. 2020 Oct 6;11:575578. doi: 10.3389/fmicb.2020.575578 (PMC7573104; doi:10.3389/fmicb.2020.575578)

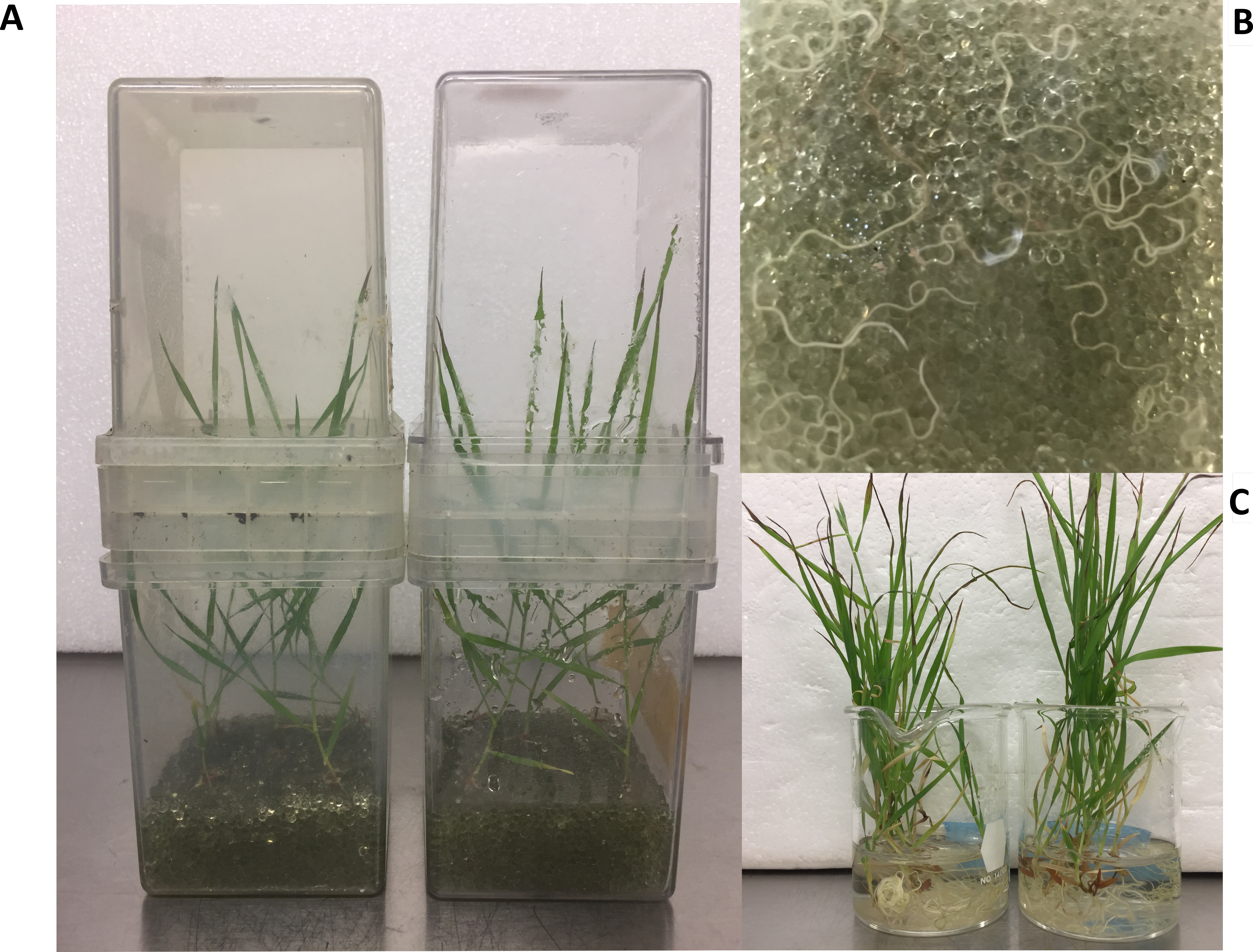

Supplement: Supplementary Figure 1 — (A) Semi-Hydroponics system in Magenta GA-7 tissue culture boxes for growing Bd21-3 under sterile conditions in glass beads saturated with 1/4th Hoagland’s solution. (B) Growth of roots in magenta boxes. (C) Root exudate collection system. [file Image_1.TIFF]

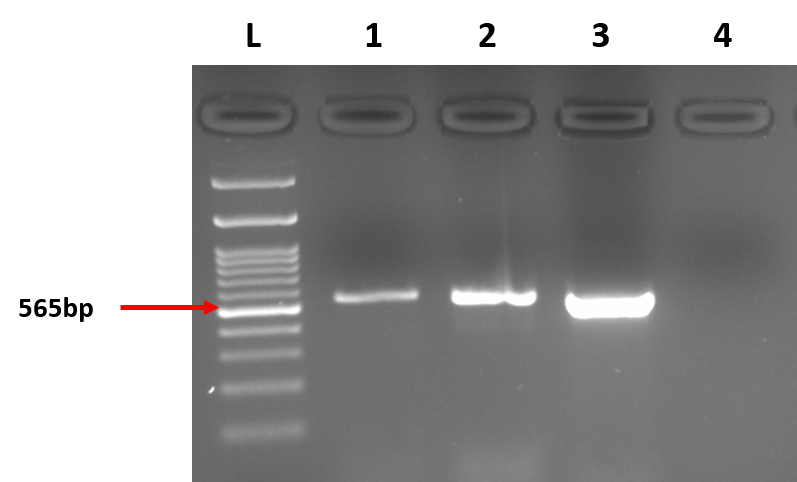

Supplement: Supplementary Figure 2 — PCR amplified products on 1% Agarose gel using specific B26 primers. L, 3Kb ladder; lane1, amplified DNA of Bd21-3 from inoculated Brachypodium roots before exudate collection. lane 2, amplified DNA product of Bd21-3 from inoculated Brachypodium roots after exudate collection. Lane 3; amplified DNA from culture of B26. Lane 4: negative control. [file Image_2.TIF]

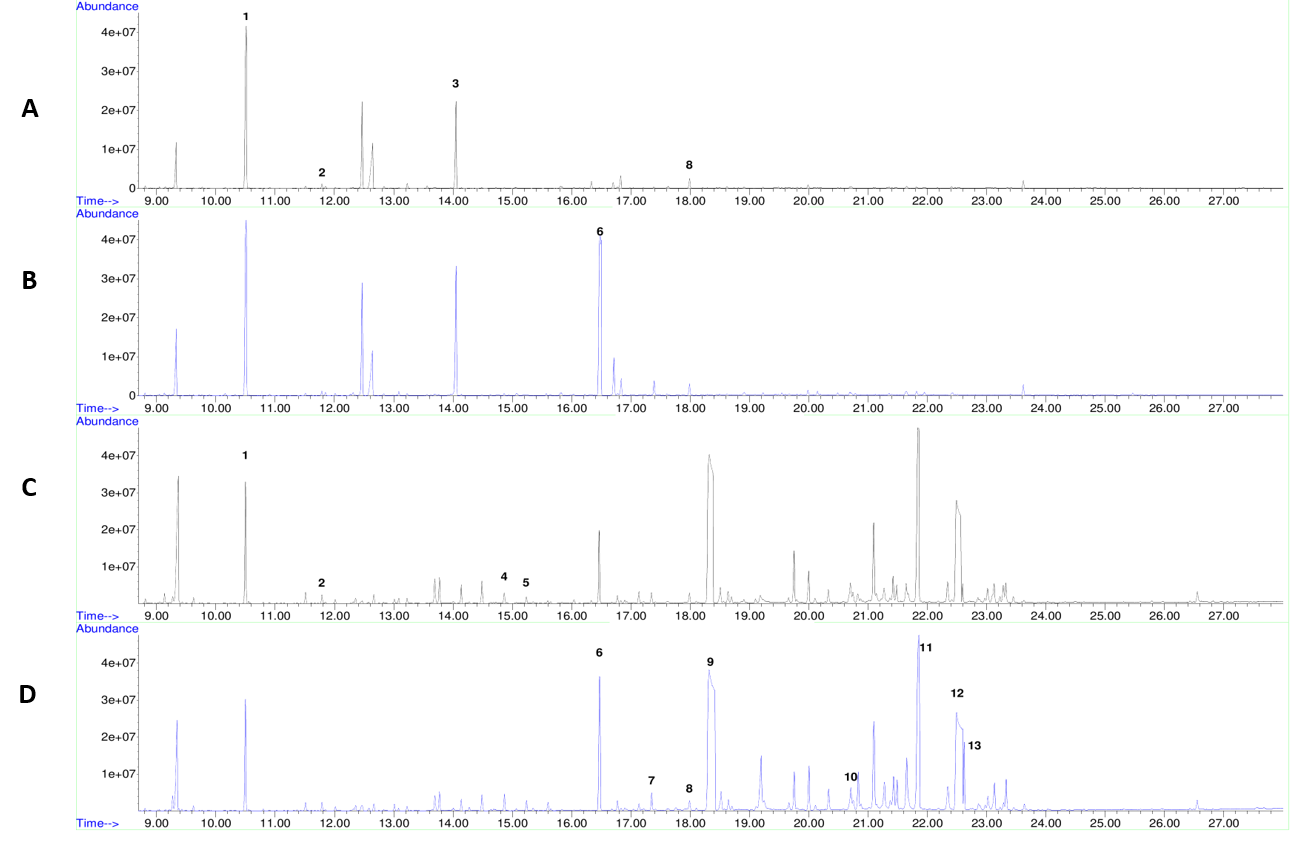

Supplement: Supplementary Figure 3 — Gas Chromatograms acquired in Scan mode for Bd21-3 samples: (A) Root Extracts Control (B) Root Extract Inoculated (C) Roots Control (D) Roots Inoculated 1, Carbonate (always present, even in blanks); 2, Lactic acid; 3, Boric; 4, Succinic; 5, Fumaric; 6, Phosphoric; 7, 2-ketoglutaric; 8, Myristic-d27 (Internal standard); 9, Malic; 10, Aconitic; 11, Dopamine; 12, Citric; 13, Isocitric. [file Image_3.tiff]
